# Chemical labelling for m^6^A detection: opportunities and challenges

**DOI:** 10.1016/j.fmre.2021.11.034

**Published:** 2021-12-24

**Authors:** Yafen Wang, Wei Yang, Xiang Zhou

**Affiliations:** College of Chemistry and Molecular Sciences, Wuhan University, Wuhan 430072, China

## Abstract

**Figure:**
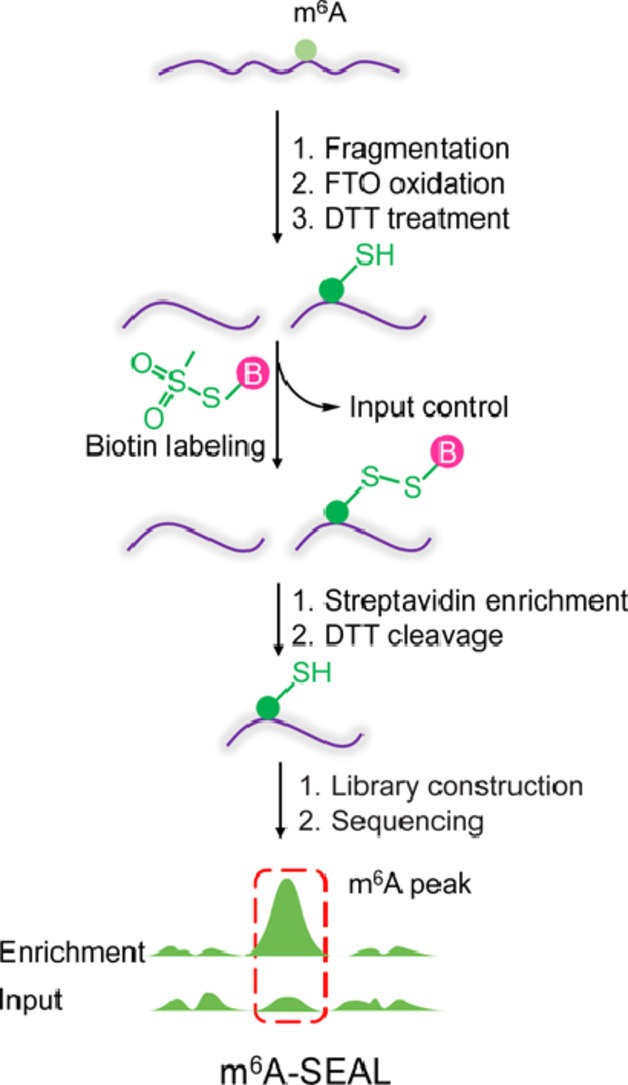

*N*^6^-methyladenosine (m^6^A) is one of the most abundant and important epigenetic modifications in eukaryotic mRNA. The finding that m^6^A is a dynamic modification based on methyltransferase and demethylase has sparked a wave of ‘epitranscriptomics’, although the term ‘epitranscriptomics’ is still controversial. With increasingly intensive research, an increasing number of functions of m^6^A have been explored, and the results have elucidated that m^6^A is involved in the splicing, nuclear export, translation, and degradation of mRNA. m^6^A also has some implications in human disease. For example, promoter-bound m^6^A methylase METTL3 maintains acute myeloid leukemia (AML) through m^6^A-dependent translation control [1]. Recent studies have also found that overexpression of METTL3 promotes the development of AML and those small molecules inhibitors of METTL3 effectively inhibit the development of AML. These results represent an encouraging step towards the development of new drugs to treat AML [2].

The identification of m^6^A is crucial for studying the function of m^6^A. The chemical inertness of m^6^A makes it difficult to label directly. However, the oxidation products during m^6^A demethylation show excellent chemical reactivity. Jia's group used the m^6^A demethylase FTO protein as a catalyst to convert chemically inert m^6^A on mRNA into the highly reactive intermediate product *N*^6^-hydroxymethyl adenine (hm^6^A). They then used the sulfhydryl group of dithiothreitol (DTT) to react with hm^6^A, which converts the unstable hm^6^A into the more stable sulfhydryl addition product dm^6^A. The free sulfhydryl groups on dm^6^A can react quickly with methanethiosulfonate (MTSEA) to produce biotin labelling at the position of m^6^A on the mRNA, which can be captured by streptavidin beads to enrich the m^6^A-containing RNA, and the captured fragments are then used for subsequent high-throughput sequencing. This method is named m^6^A-SEAL (FTO-assisted m^6^A selective chemical labelling method) (Fig. 1) [3]. This was the first study to use the FTO enzyme to assist in the realization of m^6^A chemical labelling and was successfully applied to high-throughput sequencing. m^6^A-SEAL has high sensitivity and high specificity and can be applied to low input of mRNA samples. *N*^6^,2’-*O*-dimethyladenosine (m^6^A_m_) is a modification discovered at the first nucleotide of certain mRNAs and FTO also can oxidize m^6^A_m_. The FTO oxidation conditions currently used in m^6^A-SEAL can specifically oxidize m^6^A to hm^6^A. By optimizing FTO oxidation conditions, m^6^A-SEAL can realize the labelling and sequencing of cap m^6^A_m_. m^6^A-SEAL has the following disadvantages: (1) Single-base resolution detection cannot be achieved. (2) FTO enzyme activity may vary from batch to batch, and it is difficult to control the FTO oxidation conditions. (3) It cannot quantify methylation stoichiometry. With continued optimization and modification of m^6^A-SEAL, single-base resolution detection is expected.

**Fig. 1:**
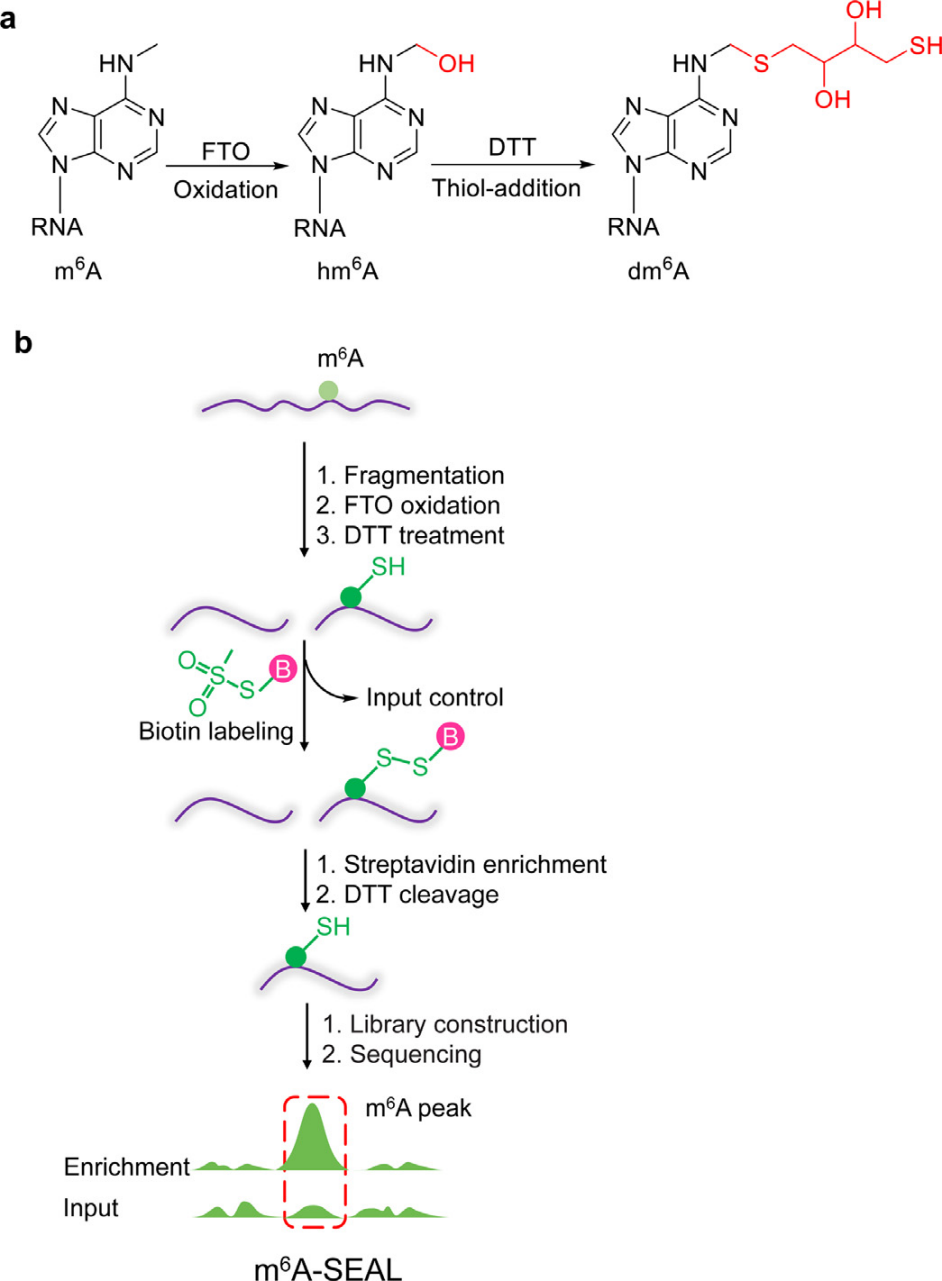
**(a) Illustration of the formation of dm^6^A. (b) Schematic diagram of m^6^A-SEAL****[3]**.

Aromatic amines can react with nitrite under acidic conditions to form nitrosamines, which are further dehydrated to form diazonium ions, and the diazonium ions are hydrolysed to obtain deamination products. The formation of diazonium ions requires additional N-H; therefore, primary aromatic amines can form diazonium ions, but secondary aromatic amines cannot. The *N*^6^ position of m^6^A is a secondary amine, and adenosine (A) is a primary amine. Inspired by the nitrite-mediated diazotization reaction of aromatic amines, NOseq was established by the deamination of A to form hypoxanthine (I) under appropriate conditions (Fig. 2) [4,5]. During the reverse transcription process, I is paired with cytosine (C) and thus read as guanine (G); that is, the treated A is mutated from A to G, whereas m^6^A is still read as A. Compared with the untreated group, m^6^A sites are those where the ratio of the A site in the reference sequence that was read as A did not decrease. Due to the low efficiency of A deamination, NOseq can effectively detect only sites with m^6^A contents higher than 50%. After enrichment with m^6^A antibody, NOseq was used to detect sites with a modification ratio lower than 10%. Current NOseq technology has the following limitations: (1) In addition to A, C and G also undergo deamination to form uracil (U) and xanthine (X), respectively. X may be read as A under certain reverse transcription conditions. These additional mutations increase the difficulty of bioinformatic analysis. (2) NaNO_2_ causes RNA degradation, which severely limits the utility of NOseq if the RNA sample quantities are low. (3) The low deamination efficiency results in the loss of some m^6^A sits with low stoichiometry. (4) m^6^A_m_ cannot be deaminated in the presence of NaNO_2_, resulting in NOseq not being able to distinguish between m^6^A and m^6^A_m_. Subsequent research can improve the sensitivity of detection and reduce the difficulty of bioinformatic analysis by further optimizing the reaction conditions, improving the deamination efficiency of A and reducing the deamination of other bases.

**Fig. 2:**
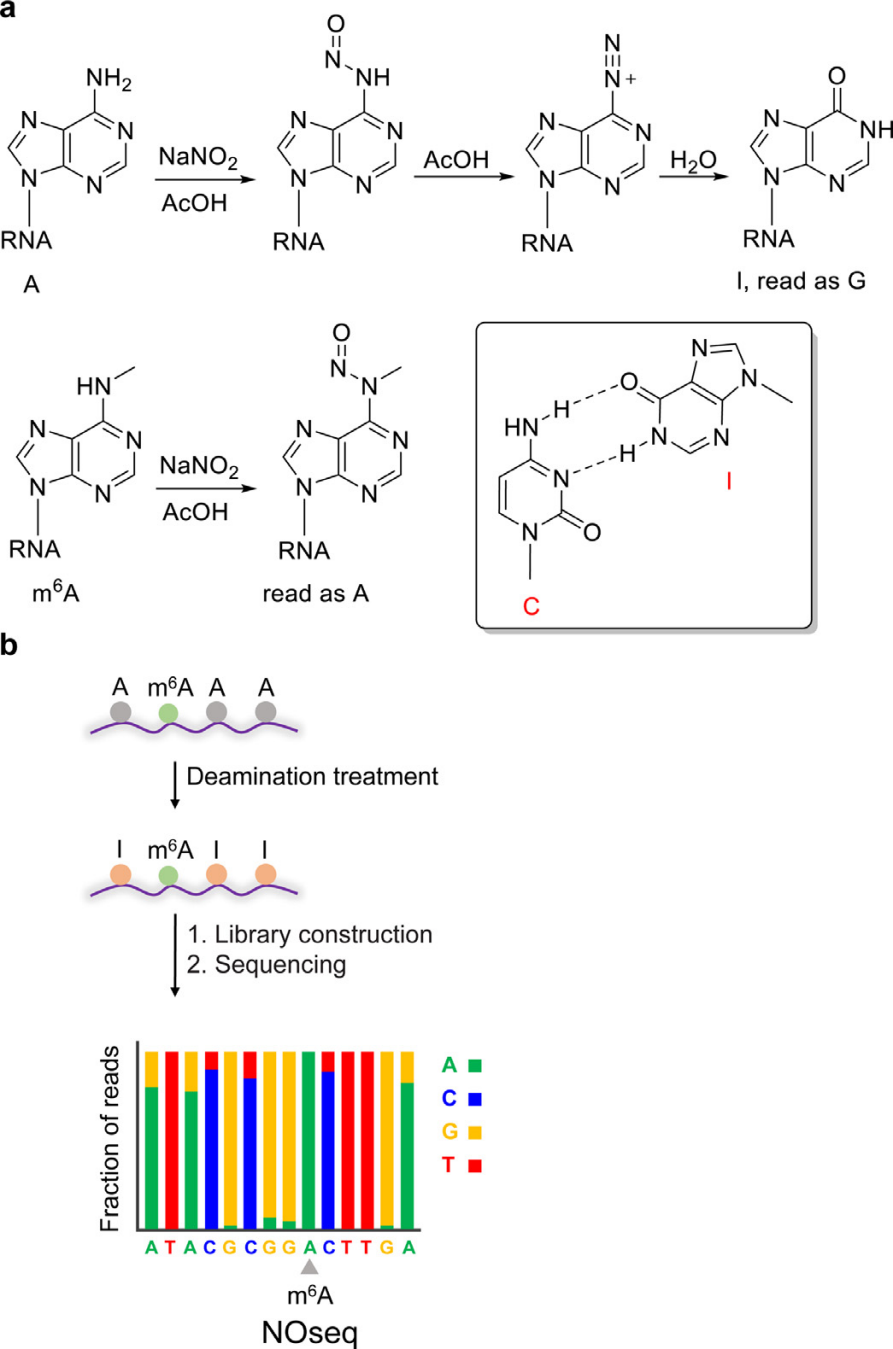
**(a) Illustration of the deamination of A. (b) Schematic diagram of NOseq****[4****,****5]**.

*S*-adenosylmethionine (SAM) is an intracellular cofactor of methyltransferase that can transfer its methyl group to specific A on mRNA under the action of mRNA m^6^A methyltransferase (METTL3/METTL14) to form m^6^A. SAM is synthesized from methionine and adenosine triphosphate (ATP) under the catalysis of methionine adenosyl methyltransferase. Liu's group labeled A with allyl-SAM (methyl is replaced by allyl), resulting in *N*^6^-allyladenosine (a^6^A), which was then iodinated to form *N*^1^,*N*^6^-cyclized A (cyc-A). The *N*^1^ position of A is involved in cyclic formation; thus, the pairing of A and thymine (T) was disturbed. The cyclic product caused misincorporation during reverse transcription and was read as T, C, or G, distinguishing a^6^A from unmodified A. Since selenium (Se) is less electronegative than sulfur, allyl-SeAM's allyl had a better leaving ability than that of allyl-SAM. They then used allyl-SeAM for metabolic labelling to detect m^6^A at the transcriptome level, termed m^6^A-label-seq (Fig. 3) [6]. When *Se*-allyl-_L_-selenohomocysteine was fed to the cells, allyl-SeAM was produced under the action of intracellular methionine adenosyl methyltransferase. Adenosines on the RNA that were modified by m^6^A methyltransferase to form m^6^A were replaced by a^6^A in the m^6^A biogenesis process. The geometrical structures of isopentenyl and allyl are very similar, so commercial *N*^6^-isopentenyladenosine (i^6^A) antibody can also be used for a^6^A enrichment. The enrichment of a^6^A-RNA reduces the sequencing cost. The single-base resolution map of m^6^A at the whole transcriptome level was obtained by the bioinformatic analysis of mutation sites. The characteristics of the sequencing method of this study are as follows: (1) the metabolic sources of methylation sites are labelled, and they are directly measured with single-base resolution, which has a higher accuracy rate than the indirect method; (2) the method is applicable to the identification of a variety of different methylation sequences (m^6^A motifs) in the internal RNA of cells; and (3) the method has advantages in determining the modification of m^6^A clusters. This method provides an important tool for the future study of m^6^A methylation sites in the cell nucleus. m^6^A-label-seq can be used for metabolic labelling of other RNA modifications. The limitation of m^6^A-label-seq is that it requires the metabolism of *Se*-allyl-_L_-selenohomocysteine and can only be applied to cellular systems. In addition, the method needs to be optimized and improved in terms of labelling yield and labelling time window scale to facilitate the study of more cellular event processes.

**Fig. 3:**
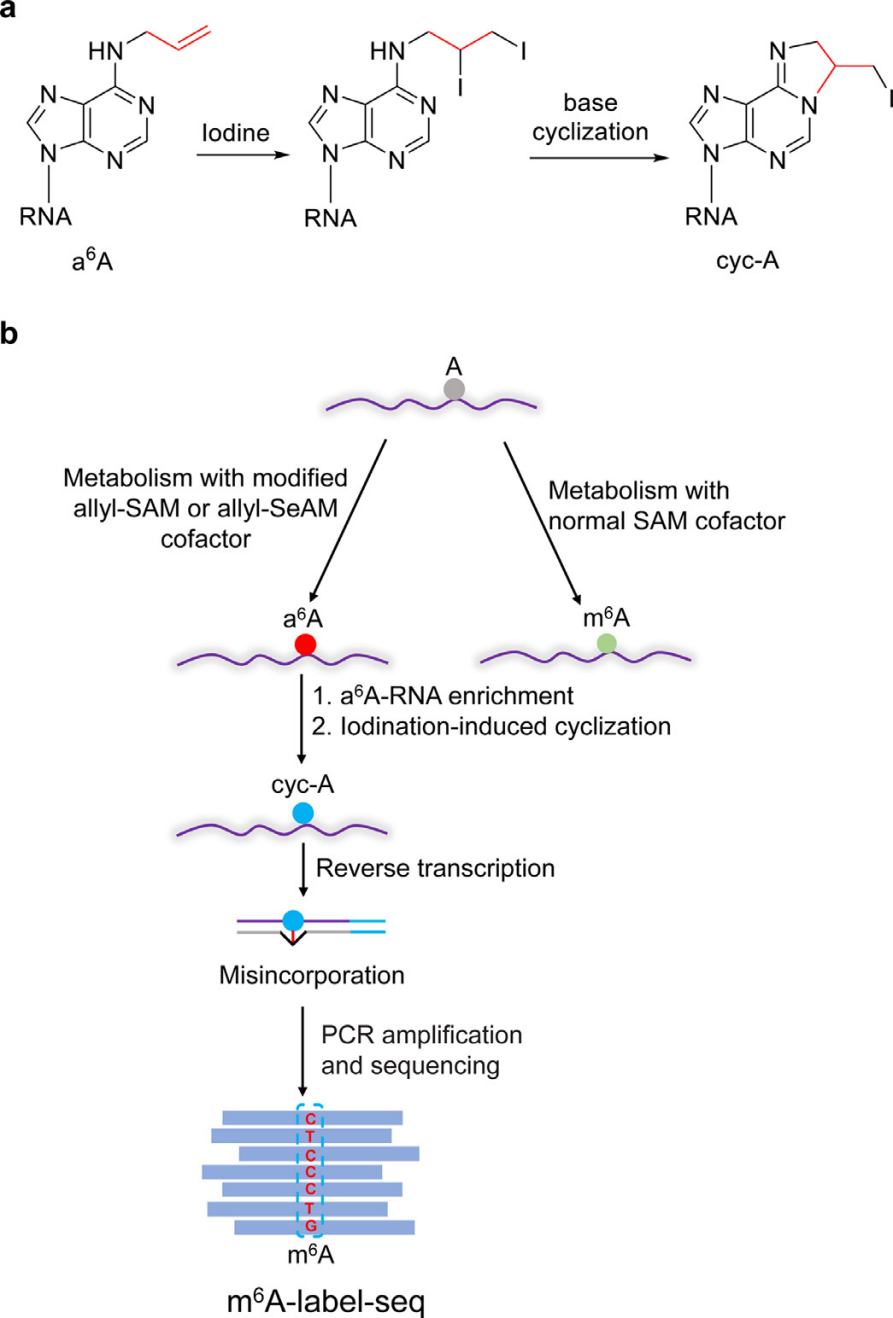
**(a) Illustration of the formation of cyc-A. (b) Schematic diagram of m^6^A-label-seq****[6]**.

Prior to the development of the above-mentioned chemical labelling strategy, the m^6^A-specific antibody enrichment strategy, MeRIP-seq, solved the main puzzle of profiling the map of m^6^A in the transcriptome. Based on MeRIP-seq, several strategies to improve resolution have emerged, such as miCLIP [7] and PA-m^6^A-seq [8]. tMeRIP-seq [9], based on transposase, can detect m^6^A in a low input sample. However, these antibody-based strategies cannot quantify m^6^A, and the lack of such information hinders the functional study of m^6^A. In addition to antibodies, there are some antibody-independent strategies such as m^6^A-sensitive endonuclease methods that can also identify m^6^A, for instance, MAZTER-seq [10] and m^6^A-REF-seq [11], which not only have the advantage of single-base resolution but also have the inability to quantify m^6^A stoichiometry. However, since endonuclease can only recognize ACA motifs, these strategies can only identify about 25% m^6^A sites, cannot recognize clustered sites and fail to distinguish between m^6^A and m6A_m_. Chemical labelling strategies can partially compensate for these deficiencies. For example, chemical labelling does not have sequence bias. The m^6^A-SEAL technique is improved so that the labelled m^6^A undergoes reverse transcription misincorporation, which may replace miCLIP.

Due to space limitations, here, we present only an overview of some current achievements in chemical labelling for m^6^A detection. As discussed, chemical labelling strategies compensate for the shortcomings of some methods, but there is still much room for improvement. The existing chemical labelling methods have the problems of insufficient labelling efficiency and low specificity (Table 1). High labelling efficiency is conducive to finding more m^6^A sites, single-base resolution feature facilitates the identification of more accurate m^6^A sites, and the strategy of specifically enriching m^6^A fragments can reduce the cost of sequencing. It is worthwhile to develop a strategy to achieve m^6^A enrichment and single-base resolution detection independent of antibodies.

**Table 1:** **A summary of the advantages and limitations of the three sequencing methods**.

Sequencing methods	Advantages	Limitations
m^6^A-label-seq	Single-base resolution, detecting clustered m^6^A sites	Requires the metabolism, applicable only to cellular systems
m^6^A-SEAL	Antibody-free enrichment, a greater specificity and sensitivity, distinguish m^6^A and m^6^A_m_ through optimizing oxidate condition	Needs to be improved to single base resolution, the enzyme activity of different batches of FTO is different, cannot quantify m^6^A
NOseq	Single-base resolution, quantification of methylation stoichiometry	Lack of specificity and sensitivity, induce RNA degradation, cannot distinguish m^6^A and m^6^A_m_

## Declaration of Competing Interest

The authors declare that they have no conflicts of interest in this work.
